# Moderated Mediation in Problematic Digital Gaming Among Adolescents: Self-Esteem, Social Anxiety, Self-Concealment, and Sensation Seeking

**DOI:** 10.3390/children12121683

**Published:** 2025-12-11

**Authors:** İbrahim Erdoğan Yayla, Samet Makas, Lokman Koçak, Murat Yıldırım, Kübra Dombak

**Affiliations:** 1 Faculty of Education, Guidance and Psychological Counselling, Sakarya University, 54050 Sakarya, Türkiye; ibrahim.yayla2@ogr.sakarya.edu.tr (İ.E.Y.); kubra.dombak1@ogr.sakarya.edu.tr (K.D.); 2 Faculty of Education, Guidance and Psychological Counselling, Bayburt University, 6900 Bayburt, Türkiye; lokmankocak@bayburt.edu.tr; 3 Faculty of Science and Letters, Department of Psychology, Agri Ibrahim Cecen University, 04100 Ağrı, Türkiye; muratyildirim@agri.edu.tr; 4 Psychology Research Center, Khazar University, Baku 1009, Azerbaijan

**Keywords:** problematic digital gaming, self-esteem, self-concealment, social anxiety, sensation seeking, adolescents, moderated mediation

## Abstract

**Background**: Problematic digital gaming among adolescents is a common behavioral issue that has negative consequences on school functioning and mental health. Theoretically, it is predicted that low self-esteem paves the way for problematic gaming by increasing social anxiety and self-concealment; this indirect pathway may be stronger in sensation seeking adolescents. **Aims**: The aim of this study is to examine the relationship between self-esteem and problematic digital gaming among adolescents, first through the PROCESS Model 6 in terms of social anxiety and self-concealment as mediators, and subsequently through Model 89 in terms of moderated mediation by sensation seeking. **Method**: The sample consisted of 448 Turkish adolescents (243 (54.2%) males, 205 (45.8%) females) aged between 13 and 18 years (M = 15.9). Participants completed the Game Addiction Scale for Adolescents—Short Form, Rosenberg Self-Esteem Scale, Self-Concealment Scale, Brief Sensation Seeking Scale and the Social Anxiety Scale-8. **Results**: A significant negative relationship was found between self-esteem and problematic digital gaming. Social anxiety and self-concealment fully mediated this relationship (indirect effects were significant). Sensation seeking conditioned the mediation by strengthening pathways to problematic digital gaming (moderated mediation was significant). The indirect effect was greater in those with high sensation seeking. **Conclusions**: The findings indicate that the negative effect, ranging from self-esteem to problematic digital gaming, progresses through social anxiety and self-concealment, and that this mediation is strengthened in adolescents with high sensation seeking. These results emphasize the need to integrate social anxiety reduction and self-expression skills into interventions in a target-sensitive manner, taking into account differences in sensation seeking.

## 1. Introduction

Advances in information and communication technologies have led to the integration of digital tools into daily life, and this trend has become particularly evident among adolescents. According to data from the Turkish Statistical Institute [1], adolescents are the group that uses technology most intensively in Turkey. Digital technologies enhance individuals’ quality of life through their multifaceted functions, including discovery, information sharing, learning, entertainment, communication, and socialization [2]. Similarly, digital games have become not only a means of entertainment for children and adolescents but also an integral part of socialization, identity construction, and the development of social belonging processes [3,4]. Easy access to mobile devices and advancements in internet infrastructure have also strengthened this interaction [2]. However, excessive and problematic use of digital technologies can trigger addictive behaviors due to their function as an escape and distraction from daily life and can negatively impact both the physical and mental health of individuals [5,6,7]. The immersive nature of games and long-term engagement, in particular, lead to problematic digital game playing behaviors [8,9].

Problematic digital gaming is defined as an individual’s inability to control their gaming behavior, their continued gaming despite adverse consequences, and the negative impact on other areas of life [10]. The American Psychiatric Association [11] discusses this behavior under the heading of “Internet Gaming Disorder,” while the World Health Organization [12] classifies it as “Gaming Disorder”. Although there is no complete consensus on diagnostic criteria, problematic gaming exhibits characteristics similar to addiction. This condition is characterized by cognitive and behavioral symptoms, including preoccupation, restlessness when unable to play, failure to control gaming time, and neglect of academic and social obligations [10]. Research shows that problematic digital game playing is positively associated with depression, anxiety, attention deficit hyperactivity disorder, obsessive–compulsive symptoms, and social isolation [13,14,15] and negatively associated with life satisfaction, self-regulation, psychological well-being, and self-esteem [16,17]. Adolescence, in particular, stands out as a developmental stage more vulnerable to problematic gaming behavior due to identity development, the search for social belonging, and emotional fluctuations [18]. Therefore, understanding the effects of gaming habits during adolescence on an individual’s psychosocial development is of great importance.

### 1.1. Self-Esteem and Problematic Digital Gaming

The transitions experienced during this period determine adolescents’ self-perception and self-esteem [19]. Self-esteem is a characteristic that emerges as a result of an individual’s self-awareness, realistic assessment of their strengths and weaknesses, and acceptance of themselves as they are, resulting in feelings of self-confidence, respect, and love [20]. High self-esteem leads to positive self-evaluations, while low self-esteem leads to feelings of worthlessness and insecurity [21]. Healthy family relationships, peer support, and social acceptance play a critical role in self-development during childhood and adolescence [6]. During this period, adolescents intensely seek social approval and peer acceptance, and can easily obtain communication and validation through digital devices [22]. However, some adolescents who struggle with identity formation may view digital environments as a means of escaping real responsibilities, making them more vulnerable to digital addiction [23].

Digital games have become an integral part of entertainment, socialization, and identity formation in the lives of adolescents today [24]. As of December 2021, 53.6% of internet users reportedly played online games [25]. The reward mechanisms, high interaction, and pleasure offered by online games make them particularly appealing to adolescents who struggle with self-control [26]. Research shows that the main factors driving adolescents to digital addiction are psychological needs such as independence, respect, and self-actualization [27]. These needs play a crucial role in the development of adolescents’ self-esteem, and deficiencies in self-esteem can increase the risk of problematic digital gaming [28]. The literature indicates a strong relationship between problematic gaming and self-esteem [29]. Low self-esteem increases the risk of addiction to online games in adolescents, while high self-esteem is seen as a protective factor against addictive behaviors [28]. Indeed, it has been reported that individuals with low self-esteem project their ideal selves onto virtual characters through gaming, but this discrepancy between the actual and ideal self-strengthens depression, social isolation, and addictive behaviors in the long term [30]. Furthermore, problematic internet and gaming use has been shown to decrease with increases in self-esteem, and strengthening self-esteem through psychosocial support has been shown to alleviate addiction symptoms [31]. In summary, strengthening self-esteem in adolescents stands out as a critical protective factor in preventing problematic digital gaming behaviors and reducing the risk of addiction.

### 1.2. Mediating Effect of Social Phobia

During adolescence, self-esteem, social relationships, and digital behaviors constitute an important area of interaction [32]. Low self-esteem reduces the individual’s participation in social environments, makes it challenging to communicate effectively with peers, and leads to anxiety in social interactions [33]. Over time, the fear of making mistakes or being negatively judged by others further restricts the adolescent’s social life and reinforces the decline in self-esteem [30]. All of these processes pave the way for the development of social phobia, characterized by social avoidance and intense anxiety in social settings [23]. Social phobia is a critical psychological variable highlighted in the relationship between self-esteem and problematic digital game playing; social phobia is defined as intense anxiety and fear in social environments where an individual may be subject to the evaluation of others [34]. This anxiety leads individuals to avoid social interactions and also leads to a decrease in self-esteem [35]. Indeed, studies show that self-esteem is a strong predictor of social phobia, with individuals with low self-esteem experiencing more social anxiety [36].

Adolescents with high social phobia may avoid face-to-face interactions while expressing themselves more comfortably in online games, which may increase problematic gaming behaviors [37]. The safe environment provided by online games through anonymity, constant feedback, and rewards creates an attractive escape for individuals with social phobia [38]. However, this can lead individuals to become addicted to digital environments rather than improving their social skills. According to the Compensatory Internet Use Theory, negative emotions such as social isolation, stress, or anxiety can lead individuals to engage in online activities, including digital games [39]. For individuals with social phobia, online games may offer a safer social environment, away from the risk of negative evaluation they face in social interactions [40]. Indeed, research indicates a positive correlation between social phobia and problematic digital gaming [38,41]. These individuals tend to turn to online environments to reduce the anxiety they experience in offline social interactions [42]. However, this tendency can cause individuals to distance themselves from real-life relationships further and develop an addiction to digital games [43]. One study reported that low self-esteem increases the likelihood of social phobia, and social phobia, in turn, drives individuals away from offline social environments and towards online gaming [41]. In this context, social phobia stands out as a central variable explaining the effect of self-esteem on problematic digital gaming.

### 1.3. The Mediating Effect of Self-Concealment

Self-esteem is directly related to an individual’s self-perception and self-worth. These characteristics can hinder individuals’ ability to express themselves adequately in social interactions and conceal their personal experiences from others. At this point, the concept of self-concealment emerges [44]. Self-concealment is defined as the habit of an individual to consciously conceal distressing personal information [45]. Individuals with low self-esteem may exhibit more self-concealment due to anxiety about social approval and fear of rejection [35]. While this behavior may seem to reduce individuals’ anxiety about negative evaluation in the short term, it deprives them of social support in the long term and increases psychological distress [44]. Thus, low self-esteem limits individuals’ social interactions through self-concealment, causing them to use avoidant coping strategies more frequently [46].

Online games, on the other hand, offer an attractive avenue for individuals with a high tendency to self-concealment [47]. The anonymity provided by games, the opportunity to construct an alternative identity, and the experience of success allow individuals with low self-esteem to project their ideal selves in the virtual environment [48]. This situation arises as a direct consequence of self-concealment behavior and, over time, can increase the risk of problematic digital gaming by fostering excessive gaming [49]. Indeed, research indicates that individuals with self-concealment behavior tend to spend more time playing online games, which can reinforce problematic behaviors [31,50]. Therefore, although the relationship between self-esteem and problematic digital game play suggests a direct interaction, self-concealment can be considered a critical mediating variable in this process [29]. Individuals with low self-esteem tend to seek ways to compensate for social needs they cannot satisfy in real life in digital environments due to their tendency to conceal themselves, which in turn facilitates the emergence of problematic gaming behaviors [51]. In summary, self-esteem appears to indirectly pave the way for gaming addiction, with self-concealment playing a mediating role in this relationship.

### 1.4. Serial Mediating Roles of Social Phobia and Self-Concealment

In light of the above theoretical discussions and existing empirical findings, self-esteem may predict problematic digital gaming behaviors through social phobia and self-concealment, respectively. According to the Cognitive Model of Social Phobia, individuals with high social anxiety constantly anticipate the possibility of negative evaluation by others, which triggers internalized protective strategies such as avoidance and self-concealment [52]. A review of the literature reveals that low self-esteem is associated with appearance anxiety and high social anxiety [53]. Furthermore, it has been suggested that higher self-esteem may be a protective factor in individuals with low social anxiety [54]. Conversely, a positive relationship between social anxiety and self-concealment behavior has also been observed [55]. In this context, it can be argued that adolescents with low self-esteem are more prone to social phobia, and social phobia, in turn, increases their self-concealment behaviors [56]. Indeed, adolescents with high social phobia conceal themselves more against the possibility of rejection, humiliation, or criticism by others, negatively impacting the quality of their social relationships [57]. Similar research has also revealed that individuals with social phobia exhibit a higher tendency to self-concealment [58]. Self-concealment refers to an individual’s concealment of negative emotions, thoughts, and experiences from their social environment, which can result in loneliness, social isolation, and weakened interpersonal relationships [59]. In the long term, this tendency can lead to individuals losing social support, increasing feelings of loneliness, and engaging in problematic online behaviors [60]. Therefore, the process that begins with low self-esteem reinforces self-concealment through social phobia, which functions as a mechanism that increases adolescents’ risk of engaging in digital environments and engaging in problematic digital gaming [50]. Therefore, it is reasonable to assume that social phobia and self-concealment may be serially mediated.

### 1.5. The Regulatory Role of Sensation Seeking

During adolescent sensation seeking emerges as a significant personality trait, reflecting individuals’ tendency to seek novelty, take risks, and pursue unusual experiences [61]. The fact that sensation seeking varies among individuals influences their preferences in many areas, including social relationships, recreational activities, risk-taking behaviors, and digital game use [62]. Individuals with higher sensation seeking behavior tend to underestimate the potential risks associated with activities they have not experienced before and are generally less anxious during this process [63]. Compared to individuals with lower sensation seeking behavior, they tend to consider the risks of the activity less [64]. Therefore, the excitement and pleasure they experience and will experience outweigh their anxiety.

Furthermore, numerous motivating factors encourage individuals to participate in recreational activities [65]. These factors include seeking excitement and adventure, engaging in exercise, pursuing personal stimulation, and taking calculated risks. Therefore, it can be argued that sensation seeking has the power to drive individuals to engage in a wide range of activities, from digital games to social interactions.

Among adolescents with low self-esteem, social phobia, and self-concealment are among the primary risk factors that increase the tendency toward digital games. In this context, digital games can provide a functional escape strategy for coping with social anxiety and concealment [29]. However, the moderating role of sensation seeking becomes increasingly important in this process. Adolescents with high levels of sensation seeking find the uncertainty, risk, competition, and intense stimulation offered by digital games more appealing, thus increasing the likelihood that social phobia and self-concealment behaviors will develop into game addiction [66,67]. In contrast, adolescents with low levels of sensation seeking find the risk and intense stimulation offered by games less appealing, and therefore, their risk of developing game addiction remains relatively low. This moderating role of sensation seeking can be explained by the subdimensions of adventure and excitement seeking, experience seeking, inability to inhibit, and distress sensitivity [68].

Adventure and sensation seeking refer to the tendency to prefer activities that involve intense stimulation and danger [56]. Adolescents high in this subscale may find the elements of risk, competition, and challenge inherent in digital games more appealing. For adolescents with a high tendency to seek new experiences, online games become appealing because they offer the opportunity to try on different identities, meet new people, and explore various worlds in virtual environments [69]. Boredom sensitivity refers to individuals’ low tolerance for routine and monotonous experiences; adolescents high in this trait easily become bored with mundane and repetitive activities, leading them to turn to digital games that offer more intense stimulation [68]. Therefore, when adolescents with low self-esteem and social phobia struggle to endure boredom in daily life, they are more likely to compensate for this lack with the instant rewards and varied experiences offered by games. The inability to restrain leads adolescents to struggle with limiting their gaming and controlling their behavior [69]. For that reason, when adolescents with high levels of inability to restrain turn to gaming to cope with emotional distress stemming from low self-esteem and social phobia, they can more easily escalate this behavior to addiction [67]. Studies have shown that adolescents with high sensation seeking behavior spend more time in gaming environments not only due to social phobia and concealment, but also due to their propensity for new and risky experiences. Hence, sensation seeking can be considered a critical regulatory factor mediating the strengthening of gaming addiction in a process that begins with low self-esteem and progresses through social phobia and concealment.

### 1.6. Present Study

The increasing prevalence of digital games in adolescents’ daily lives brings new areas of risk to their developmental processes. Intensive gaming behavior is associated with numerous problems, such as decreased academic performance, weakened social relationships, difficulties in emotion regulation, and impairment in daily functioning [13,14,15,70]. In this context, problematic digital gaming is considered a significant developmental risk factor for adolescents. The literature reveals that self-esteem stands out as a fundamental psychological variable in this process, and that adolescents with low self-esteem are more vulnerable to negative emotional experiences such as social anxiety and loneliness [71,72]. In particular, social phobia, as a reflection of low self-esteem, can lead adolescents to limit themselves in interpersonal relationships and resort to self-concealment behaviors [72].

**Figure 1:**
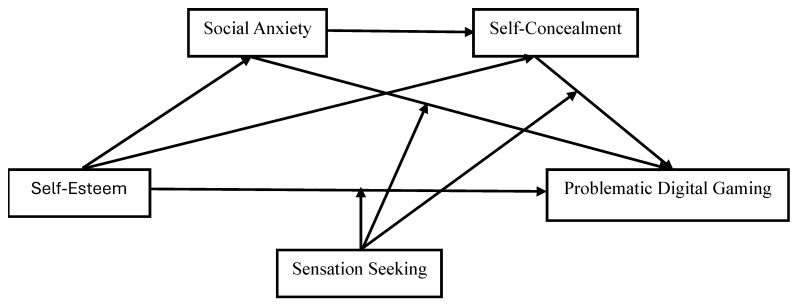
The proposed hypothetical model.

Adolescence is also a period characterized by intense sensation seeking. Adolescents with a high novelty and risk-seeking tendency tend to engage in gaming more readily due to the competition, uncertainty, and stimulus intensity inherent in digital games [73]. Within this framework, the current study aims to examine the mediating role of social phobia and self-concealment behavior, as well as the moderating role of sensation seeking, in the relationship between self-esteem and problematic digital game playing among adolescents. By considering these variables within the same model, the study aims to holistically assess the cognitive, emotional, and personality-based processes that explain problematic digital game playing. Thus, it provides a theoretical basis for early intervention and preventive strategies for digital game addiction, shedding light on the development of applicable psychoeducational programs, particularly for families, teachers, and mental health professionals. Furthermore, testing the mechanisms of “social phobia, self-concealment, and sensation seeking,” which have been studied together in limited quantities in the literature, within the same model makes the study unique. The proposed hypothetical model is shown in Figure 1.

**H1.**  *Self-esteem is negatively associated with problematic digital gaming.*

**H2.**  *Social anxiety mediates the relationship between self-esteem and problematic digital gaming.*

**H3.**  *Self-concealment mediates the relationship between self-esteem and problematic digital gaming.*

**H4.**  *Social anxiety and self-concealment chain mediate the relationship between self-esteem and problematic digital gaming.*

**H5.**  *Sensation seeking moderates [strengthens] the effects of self-esteem, social anxiety, and self-concealment on problematic digital gaming.*

## 2. Materials and Methods

### 2.1. Participants and Procedure

The study was conducted on 448 adolescents. A total of 243 [54.2%] of the participants were male, while 205 [45.8%] were female. The ages of the participants ranged from 13 to 18, with a mean age of 15.9 [SD = 1.09] years. The research was conducted in accordance with the Helsinki Declaration and relevant national guidelines. Approval for the study was obtained from a university’s Scientific Research and Ethics Committee [Date: [25 July 2025]; Decision No: [396]]. Schools with established collaboration and easy access were selected prior to implementation. Subsequently, an informed consent form explaining the purpose of the study, the principle of voluntariness, confidentiality, and the right to withdraw was sent online to the parents/caregivers of students attending these schools; children from consenting families were invited to participate in the study. Students were informed using an age-appropriate assent form before the survey and were told that they could withdraw at any time without penalty. The online data collection process was completed in one week.

### 2.2. Measures

**The Game Addiction Scale for Adolescents-Short Form**: To determine the levels of problematic use in adolescents’ digital [computer, phone, tablet, etc.] gaming behavior, Anlı and Taş [74] developed a scale. The scale consists of a single dimension and 9 items. It also has a Likert structure rated from 1 to 5. High scores indicate an increase in the level of problematic use. The fit indices for the scale in this study are χ^2^/df = 3.29, AGFI = 0.93, CFI = 0.97, GFI = 0.96, SRMR = 0.03, and NFI = 0.96. The reliability value in this study is α = 0.91.

**Self-Concealment Scale**: The scale measures adolescents’ tendency to hide information from others that they perceive as negative, distressing, or private. The scale [Original: Larson and Chastain [75], Turkish: Çok and Deniz [76]] consists of ten items, a 5-point Likert scale and a single dimension. High scores on the scale indicate an increase in adolescents’ level of self-concealment. Confirmatory factor analysis [CFA] showed good fit in the sample of this study [χ^2^/df = 2.74, RMSEA = 0.06, AGFI = 0.93, CFI = 0.97, SRMR = 0.03, GFI = 0.96]. The Cronbach’s Alpha value for the scale in the current sample is 0.91.

**Social Anxiety Scale-8**: The scale was selected to measure the levels of social anxiety experienced by adolescents. Scale [Original: Nunes et al. [77], Turkish: Can and Bozgün [78]], a 5-point Likert scale, 12 items, 3 factors [fear of negative evaluation, social avoidance and distress in general situations, social avoidance and distress in new situations]. Higher scores indicate greater social anxiety. In the study sample, Cronbach’s alpha coefficient was 0.93 for the entire scale. The results of the DFA analysis of the scale for this study indicate that the general fit indices [χ^2^/df = 2.44; GFI = 0.95; NFI = 0.96; SRMR = 0.03; TLI = 0.97; CFI = 0.98; RMSEA = 0.05] are adequate.

**Brief Sensation Seeking Scale**: The scale was used to measure the levels of sensation seeking in adolescents’ lives. Scale [Original: Stephenson et al. [79], Turkish: Çelik [80] is a 4-point Likert scale, 4 items and a single dimension. Higher scores indicate greater sensation seeking. In the study sample, Cronbach’s alpha coefficient was 0.82 for the entire scale. The results of the DFA analysis of the scale for this study indicate that the general fit indices [χ^2^/df = 1.32; GFI = 0.99; AGFI = 0.98; SRMR =0.01; TLI = 0.99; CFI = 0.99; RMSEA = 0.02] are adequate.

**Rosenberg Self-Esteem Scale**: The scale was used to measure the levels of self-esteem in adolescents’ lives. The scale [Original: Rosenberg et al. [81], Turkish: Çuhadaroğlu [82]] was a 4-point Likert scale, with 10 items and two dimensions. Articles 1, 2, 4, 6, and 7 are reverse-coded. Higher scores indicate greater self-esteem. In the study sample, Cronbach’s alpha coefficient is 0.90 and 0.87 for the subscales. The results of the DFA analysis of the scale for this study indicate that the general fit indices [χ^2^/df = 2.36; SRMR = 0.03; GFI = 0.96; TLI = 0.98; AGFI = 0.94; CFI = 0.98; RMSEA = 0.05] are adequate.

### 2.3. Data Analysis

SPSS version 27.0 was used for data analysis. First, descriptive statistics were reported. Second, Pearson Correlation analysis was used to reveal the relationships between variables. In the third step, the serial mediating effects of social anxiety and self-concealment between self-esteem and problematic digital gaming were revealed using Hayes Model 6. Model 6 was chosen because it allows for testing serial mediation effects among variables and is consistent with the theoretical framework of the study [83]. At this stage, to assess the significance of mediation effects, the bootstrap method involving 5000 repeated resamples was used to obtain 95% confidence intervals, thereby enhancing robustness and prediction accuracy. Finally, whether the serial mediating effects of social anxiety and self-concealment were moderated by sensation seeking was tested using Hayes Model 89. Model 89 allows for the examination of conditional indirect effects based on the assumption that the level of intermediary relationships may vary according to individual differences; in this respect, it is theoretically consistent with the interactive dynamics predicted in the research model [83].

## 3. Results

### 3.1. Preliminary Analyses

Examining Table 1 reveals that the skewness values range from −0.29 to 0.62, and the kurtosis values range from −1.11 to −0.79. These values, which fall between +1.5 and −1.5, are within the acceptable range for normality. No problematic multicollinearity was observed among the variables: VIF = 1.12–1.48, tolerance = 0.67–0.89, and condition index = 5.75–14.94 values, VIF < 10, CI < 30, and tolerance > 10 thresholds, meeting the acceptance criteria [84].

Table 1 shows that problematic digital gaming is negatively correlated with self-esteem [r = −0.20, *p* < 0.001], is positively correlated with social anxiety [*r* = 0.46, *p* < 0.001], self-concealment [*r* = 0.46, *p* < 0.001] and sensation seeking [*r* = 0.61, *p* < 0.001]. Self-esteem is negatively related to social anxiety [*r* = −0.23, *p* < 0.001], self-concealment [*r* = −0.26, *p* < 0.001] and sensation seeking [*r* = −0.01, *p* > 0.001]. Social anxiety is positively related to self-concealment [*r* = 0.49, *p* < 0.001] and sensation seeking [*r* = 0.44, *p* < 0.001]. Self-concealment is positively related to sensation seeking [*r* = 0.42, *p* < 0.001].

### 3.2. Testing for the Serial Indirect Effects of Self-Esteem and Problematic Digital Gaming

The results of Hayes’ [83] Process Macro [Model 6], which examined the serial mediating effect of social anxiety and self-concealment on the relationship between self-esteem and problematic digital gaming, are presented in Table 2.

**Figure 2:**
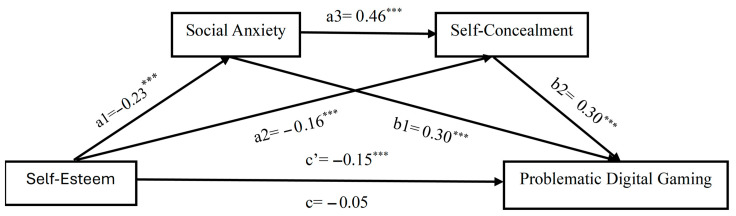
Results of the serial mediation test. *** *p* < 0.001.

When Table 2 and Figure 2 are examined together, hypothesis H1 is confirmed. In other words, self-esteem was negatively associated with problematic digital gaming [β = −0.20, *p* < 0.001]. Self-esteem was found to contribute negatively to social anxiety [β = −0.23, *p* < 0.001], and social anxiety had a positive effect on problematic digital gaming [β = 0.30, *p* < 0.001]. This confirms hypothesis H2, that social anxiety plays a mediating role between self-esteem and problematic digital gaming. Furthermore, while self-concealment is negatively affected by self-esteem [β = −0.16, *p* < 0.001], problematic digital gaming has a positive effect [β = 0.30, *p* < 0.001]. Thus, self-concealment plays a mediating role between self-esteem and problematic digital gaming, and hypothesis H3 is also confirmed.

Looking at the findings for H4 hypothesis, the direct effect of self-esteem on problematic digital gaming is not significant [β = −0.051, 95% CI = [−0.1526, 0.0364]]. In contrast, the total indirect effect is significant [B = −0.146, 95% CI = [−0.1965, −0.0977]]. Furthermore, the confidence intervals for each of the three indirect paths do not include 0 [self-esteem → social anxiety → problematic digital gaming; self-esteem → self-concealment → problematic digital gaming; self-esteem → social anxiety → self-concealment → problematic digital gaming]. When mediators were included in the model, the direct effect of self-esteem on problematic digital gaming lost its significance. This finding indicates that the effect of self-esteem is largely mediated by social anxiety and self-concealment variables. These findings indicate full mediation in the current relationship and confirm hypothesis H4. Details are presented in Table 3.

### 3.3. Testing for the Moderation Effect of Sensation Seeking

The moderated serial mediation Hayes PROCESS model 89 was tested with sensation seeking included as a moderator variable, and the results are presented in Table 4. When examining the regression model, the interaction terms were found to be significant because their confidence intervals did not include 0. The interaction between sensation seeking and self-esteem [β = −0.1315, 95% CI = [0.2250, 0.0364]] is negative, while the interactions with social anxiety [β = 0.1276, 95% CI = [0.0584, 0.1969]] and self-concealment [β = 0.1139, 95% CI = [0.0261, 0.2017]] are positive. The overall regression model is statistically significant and explains 51.3% of the variance in problematic digital gaming [R^2^ = 0.5126], [F = 66.10, *p* < 0.001].

**Figure 3:**
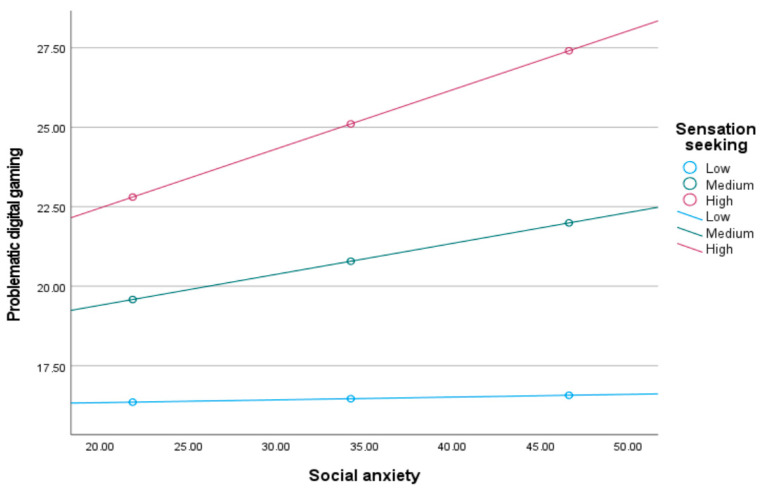
Sensation seeking moderated the relation between social anxiety and problematic digital gaming.

To explain the three interactions mentioned above in greater depth, a simple slope analysis was performed. It was revealed how the relationships changed when the sensation seeking tendency was low/medium/high [M ± 1 SS]. The relationship between low sensation seeking and problematic digital gaming with social anxiety was insignificant [β = −0.0032, 95% CI = [−0.0232, 0.0173]], while it was significant at the medium [β = −0.0354, 95% CI = [−0. 0668, −0.0112]], and high [β = −0.0675, 95% CI = [−0.1212, −0.0265]] levels of sensation seeking. Figure 3 shows that the relationship between social anxiety and problematic digital gaming strengthens as sensation seeking increases.

**Figure 4:**
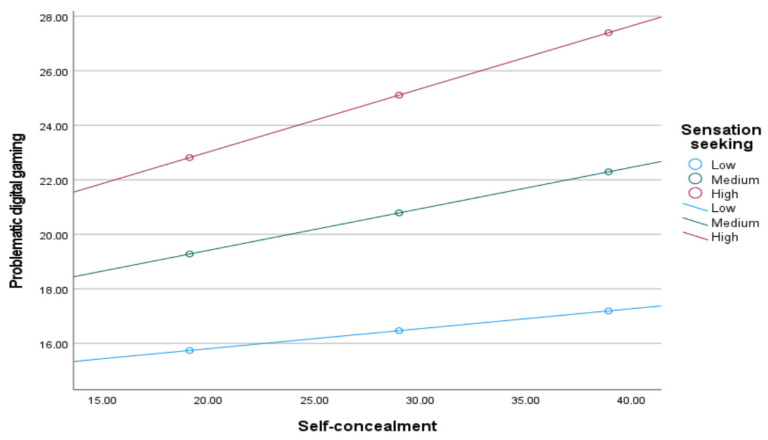
Sensation seeking moderated the relation between self-concealment and problematic digital gaming.

The relationship between self-concealment and problematic digital gaming is β = −0.0151, β = −0.0315, and β = −0.0478, respectively, at a 95% confidence interval that does not include 0 when the sensation seeking is low, medium, and high. Figure 4 shows that the relationship between self-concealment and problematic digital gaming strengthens as the sensation seeking increases.

**Figure 5:**
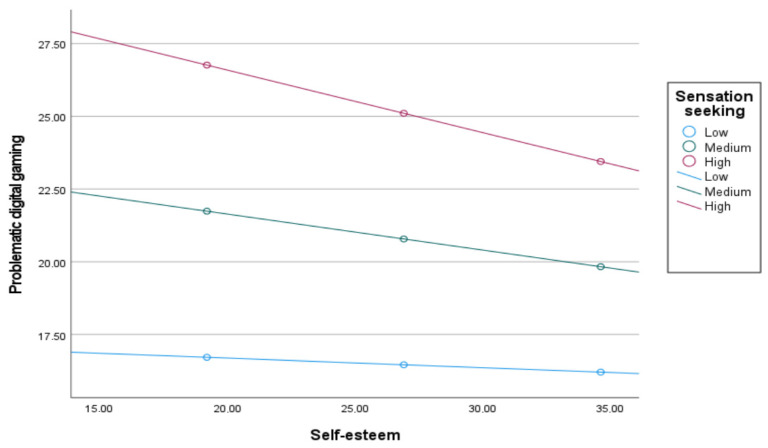
Sensation seeking moderated the relation between self-esteem and problematic digital gaming.

The serial mediating relationship between self-esteem and problematic digital gaming when the sensation seeking is low, medium, and high is [β = −0.0097], [β = −0.0202], and [β = −0.0306], respectively, at a 95% confidence interval not including 0. Figure 5 shows that as the search for sensation seeking increases, the relationship between self-esteem and problematic digital gaming strengthens. Table 5 shows that the effect in the three paths mentioned above increases with the increase in sensation seeking tendency, confirming hypothesis h5.

## 4. Discussion

The findings of this study reveal that self-esteem is negatively associated with problematic digital gaming behavior in adolescents. This result parallels meta-analytic studies showing that low self-esteem is a significant risk factor for types of digital addiction [29]. Similarly, Widyanto and Griffiths [31] also reported that self-esteem is a protective factor against problematic internet use. Therefore, it can be said that adolescents’ self-perceptions play a critical role in understanding risky behavior patterns in digital environments.

One of the study’s key findings is that social anxiety mediates the relationship between self-esteem and problematic digital gaming behavior. The effect of social anxiety on internet and gaming addiction has been frequently reported, particularly among adolescents [29,42]. For example, Cruz et al. [71] revealed the negative cycle between social anxiety and self-esteem during the pandemic. Current findings show that social anxiety provides an important psychosocial basis that increases the tendency to engage in online gaming. However, the study also found that self-concealment plays a mediating role in the relationship between self-esteem and problematic digital gaming. Self-concealment is characterized by individuals internalizing their negative emotions rather than sharing them, and is associated with addictive behaviors [35,48]. Jin et al. [58] also confirmed the relationship between self-concealment tendencies and social appearance anxiety, and negative psychological outcomes. In this context, the current study demonstrates that self-concealment serves as an important bridge in the pathway to problematic gaming behavior and, together with social anxiety, creates a serial mediating effect, offering a multi-layered mechanism for the transformation of low self-esteem into problematic gaming behavior.

One of the original contributions of this study is the confirmation of the regulatory role of sensation seeking. The findings reveal that in adolescents with high sensation seeking, self-esteem, social anxiety, and self-concealment processes more strongly predict problematic gaming behavior. This result is consistent with the literature showing the facilitating role of sensation seeking in risky behaviors [66,74] and is also supported by Zuckerman and Aluja’s [68] theory. Furthermore, it is consistent with findings that the speed, competition, and reward mechanisms offered by games reinforce the risk of addiction in individuals with high sensation seeking [26]. These findings are consistent with theoretical frameworks explaining problematic gaming behavior. In particular, the I-PACE model and compensatory use theories emphasize that low self-esteem and high anxiety levels may increase the tendency toward online behavior [18]. Furthermore, studies showing that escape-oriented use in adolescents are associated with addiction [22,46] support this study’s serial mediation finding. Therefore, it can be said that the chain of low self-esteem–social anxiety–self-concealment, combined with inadequate coping mechanisms and emotional regulation problems, reinforces problematic gaming behavior.

Environmental factors also play an important role in this process. The reward architecture of digital games, particularly variable-ratio reinforcement loops, challenge tasks, and social prestige elements, provides powerful triggers that convert individual sensitivities into behavior. For example, it has been reported that rewarding game elements increase problematic usage behaviors [26] and that mechanisms such as loot boxes lay the groundwork for addiction [27]. Furthermore, longitudinal findings have shown that online communities can increase the risk of problematic use over time [51]. In this context, the heightened sensitivity of adolescents with high sensation seeking tendencies to reward-dense environments can be considered a strong mechanism explaining our moderation finding.

Clinical and epidemiological data also support the findings of this study. It has been reported that problematic gaming behavior in adolescents often co-occurs with psychiatric comorbidities such as anxiety, depression, and attention deficit disorder and leads to serious limitations in functioning [13,14]. Furthermore, national-level data indicate that problematic gaming behavior can vary across cultures [8]. Therefore, although limiting the study to data obtained from Turkish adolescents restricts the generalizability of the findings to different socio-cultural contexts, it presents an important opportunity for designing culturally specific intervention programs [22].

From a developmental perspective, adolescence is a period characterized by increased risk-taking tendencies and an immature capacity for self-regulation [32]. In this context, problematic gaming behavior should be viewed not only as a result of individual differences but also as a consequence of developmental vulnerabilities. It can be argued that mechanisms operating along the axes of self-esteem, social anxiety, and self-concealment can negatively affect not only gaming behaviors but also adolescents’ academic achievement [75] and psychosocial adjustment.

In conclusion, this study has made original contributions to the literature by revealing multi-layered mediating and regulatory mechanisms in explaining problematic digital gaming behavior. The findings clarify how risk factors and individual differences interact in adolescents and shed light on the design of more targeted preventive and intervention programs. Another contribution of this research to the literature is its demonstration that self-concealment plays a significant mediating role on the path to problematic gaming behavior, and that, together with social anxiety, it creates a serial mediating effect, suggesting a multidimensional mechanism in the transformation of low self-esteem into problematic gaming behavior. Furthermore, the study’s finding that the chain of low self-esteem, social anxiety, and self-concealment, combined with inadequate coping mechanisms and emotion regulation problems, further exacerbates problematic gaming behavior, is another contribution to the literature. In conclusion, the current study, which provides important findings regarding the underlying causes of problematic gaming, a major global problem today, and its relationship with psychological factors, will make significant contributions to the literature.

## 5. Limitations and Conclusions

This study has some limitations. First, the research is based on a cross-sectional design. Therefore, it is not possible to draw definitive conclusions about causal relationships between variables. Future studies using longitudinal or experimental designs could more clearly examine the direction and dynamics of relationships between variables over time [6]. Another limitation is that the data were collected through self-report scales. This may have been influenced by participants’ social desirability tendencies or respondent bias. The use of observation, qualitative interviews, or multiple data collection methods is recommended in future research. Another limitation of the study is that the sample consisted solely of Turkish adolescents. This limits the generalizability of the findings to different cultural contexts. Cultural values, family structure, social media usage practices, and gaming culture may vary from country to country. For example, while individualism is at the forefront in Western societies, social anxiety, self-concealment, and motivations for gaming may differ in collectivist societies such as Turkey [71]. Therefore, it is recommended that future studies conduct cross-cultural comparative research. Research conducted in different cultural contexts can reveal whether the effects of variables such as self-esteem, social anxiety, and self-concealment on gaming behavior are universal or culture-specific. Furthermore, comparing findings across different cultures will also contribute to the cultural adaptation of preventive programs to be developed. Finally, the study only examined specific variables [self-esteem, social anxiety, self-concealment, and sensation seeking]. However, previous studies have shown that variables such as loneliness, social support, emotional regulation difficulties, and escapism may also be related to problematic digital gaming behavior [22,76]. Including these variables in future studies may provide a more comprehensive explanation.

Based on the findings of the study, several recommendations can be made. First, psycho-educational content should be developed within school-based prevention programs to strengthen adolescents’ self-esteem and reduce their social anxiety [5]. Additionally, psychological counseling programs that support emotional sharing and open communication may be beneficial for adolescents with a high tendency to hide themselves. On the other hand, it is important to identify adolescents with a high level of sensation seeking as a risk group and to expand screening studies targeting this group. Considering that adolescents with these characteristics are more sensitive to stimuli and rewards in digital gaming environments, developing alternative leisure activities and social activities for them may contribute to the prevention of risky behaviors [66,74]. Considering the limitations of future research, it is recommended that this model be tested in different cultural contexts, with different age groups, and using different methods.

### Implications

In conclusion, this study has presented a comprehensive model incorporating multiple mediating and moderating variables to explain problematic digital gaming behavior, thereby making significant contributions to the literature. The research findings highlight the necessity of multi-layered and targeted interventions to reduce problematic digital gaming behavior among adolescents. School-based programs should incorporate activities that enhance self-esteem, cognitive behavioral skills targeting social anxiety [exposure, cognitive restructuring, social skills training], and self-expression/emotional openness modules. In clinical and counseling practices, screening protocols [using short scales] can be supplemented with the simultaneous assessment of the self-esteem–social anxiety–self-concealment triad; interventions can also be structured following the sequence indicated in serial mediation [first regulating anxiety, then openness and emotion sharing]. Young people who seek high excitement are a risk group; for this subgroup, safe activities that provide alternative stimulation [sports, performing arts, maker workshops], digital literacy programs with gamified but healthy reward cycles, and short, intensive sessions increase the potential for effectiveness. At the family level, home media rules, joint problem-solving, and emotional communication skills should be supported.

At the policy and ecosystem level, redesigning digital services offered to young people with safety principles [e.g., usage limits, break/consistency reminders, transparent reward-punishment mechanisms] can reduce designs that exploit the search for high stimulation. Early warning and referral lines established through school-health-NGO collaborations enable intervention before risky patterns become entrenched. Program content should be designed with cultural sensitivity; messages should be adapted considering collective values, family, and peer norms in the Turkish context. Ultimately, the study shows that addressing risk [low self-esteem, social anxiety, self-concealment] and protective/balancing factors within the same model strengthens both clinical decision-making and educational policies in a targeted and measurable way.

## Figures and Tables

**Table 1:** The results of the correlation analysis and descriptive statistics.

Variable	1.	2.	3.	4.	5.
1. Problematic Digital Gaming	1				
2. Self-Esteem	−0.20 **	1			
3. Social Anxiety	0.46 **	−0.23 **	1		
4. Self-Concealment	0.46 **	−0.26 **	0.49 **	1	
5. Sensation Seeking	0.61 **	−0.01 **	0.44 **	0.42 **	1
Mean	21.57	26.91	34.22	28.99	10.54
SD	8.83	7.70	12.39	9.90	3.39
Condition Index	1	5.75	9.18	9.32	14.94
VIF	-	1.12	1.48	1.48	1.36
Tolerance	-	0.89	0.68	0.67	0.73
Skewness	0.62	−0.29	0.14	0.05	−0.23
Kurtosis	−0.79	−1.02	−1.11	−0.99	−1.11

** *p* < 0.01.

**Table 2:** Testing the serial mediation effect of self-esteem on problematic digital gaming.

Predictors	Model 1 [SA]		Model 2 [SC]		Model 3 [PDG]	
	*Β*	*t*	*β*	*t*	*β*	*t*
Self-esteem	−0.23	−4.90 ***	−0.16	−3.86 ***	−0.06	−1.21 ***
Social Anxiety [SA]			0.46	10.95 ***	0.30	6.38 ***
Self-Concealment [SC]					0.30	6.37 ***
R^2^	0.05		0.27		0.28	
F	24.02 ***		81.10 ***		58.25 ***	

n = 448. *** *p* < 0.001.

**Table 3:** Direct, indirect, and total effects of self-esteem on problematic digital gaming [Bootstrap 95% CIs].

Path	Effect Size	Boot SE	Boot LLCI	Boot ULCI
Direct effect	−0.0507	0.0481	−0.1526	0.0364
Indirect effect	−0.1461	0.0250	−0.1965	−0.0977
Self-esteem → social anxiety → problematic digital gaming	−0.0671	0.0213	−0.1120	−0.0300
Self-esteem → self-concealment → problematic digital gaming	−0.0481	0.0195	−0.0929	−0.0156
Self-esteem → social anxiety → self-concealment → problematic digital gaming	−0.0308	0.0087	−0.0494	−0.0154
Total effect	−0.1967	0.0532	−0.3301	−0.1209

**Table 4:** Testing the moderated mediation effect of self-esteem on problematic digital gaming.

Variables	*β*	SE	T	Bootstrap 95%CI	
				Lower	Upper
Sensation Seeking	2.0952	2.0872	1.0039	−2.0068	6.1972
Self-esteem	0.1388	0.0988	1.4042	−0.0555	0.3330
self-esteem × sensation seeking	−0.1315	0.0476	−2.7630 **	−0.2250	−0.0380
social anxiety	−0.1577	0.0754	−2.0915 *	−0.3059	−0.0095
social anxiety × sensation seeking	0.1276	0.0352	3.6228 ***	0.0584	0.1969
self-concealment	−0.0753	0.0938	−0.8035	−0.2596	0.1089
self-concealment × sensation seeking	0.1139	0.0447	2.5501 *	0.0261	0.2017
R^2^	0.5126				
F	66.0976 ***				

* *p* < 0.05, ** *p* < 0.01, *** *p* < 0.001.

**Table 5:** The indirect paths between the variables.

Path	Moderating Variable	Effect Size	Boot SE	Boot LLCI	Boot ULCI
Self-esteem → social anxiety → problematic digital gaming	Low sensation seeking	−0.0032	0.0100	−0.0232	0.0173
	Medium sensation seeking	−0.0354	0.0142	−0.0668	−0.0112
	High sensation seeking	−0.0675	0.0243	−0.1212	−0.0265
Self-esteem → self-concealment → problematic digital gaming	Low sensation seeking	−0.0151	0.0088	−0.0356	−0.0013
	Medium sensation seeking	−0.0315	0.0135	−0.0618	−0.0098
	High sensation seeking	−0.0478	0.0210	−0.0961	−0.0142
Self-esteem → social anxiety → self-concealment → problematic digital gaming	Low sensation seeking	−0.0097	0.0051	−0.0208	−0.0011
	Medium sensation seeking	−0.0202	0.0072	−0.0371	−0.0083
	High sensation seeking	−0.0306	0.0114	−0.0574	−0.0125

## Data Availability

The data presented in this study are available on request from the corresponding author. The data are not publicly available due to privacy and ethical reasons.
